# Qi Ling decoction enhances abiraterone treatment via suppression of autophagy in castration resistant prostate cancer

**DOI:** 10.18632/aging.204427

**Published:** 2022-12-19

**Authors:** Yigeng Feng, Hongwen Cao, Zixi Song, Lei Chen, Dan Wang, Renjie Gao

**Affiliations:** 1 Surgical Department I (Urology Department), Longhua Hospital Shanghai University of Traditional Chinese Medicine, Xuhui 200032, Shanghai, China

**Keywords:** Qi Ling decoction, prostate cancer, castration-resistance, tumor

## Abstract

Abiraterone acetate has exhibited impressive results in improving progression-free survival of patients with metastatic castration-resistant prostate cancer. However, many patients may develop abiraterone resistance with a variable duration of response. Hence, identifying a remedy to overcome abiraterone resistance is critical for patients with castration-resistant prostate cancer. In this study, we aim to explore the potential of Qi Ling decoction (QLD), a traditional Chinese medicine, in attenuating abiraterone resistance in prostate cancer. Cell viability and apoptosis were respectively measured by Cell Counting Kit-8 (CCK-8) assay and flow cytometry. The protein levels were assessed by Western blotting assay. Autophagosome formation was quantified by counting LC3 puncta. We found that QLD was capable of promoting abiraterone-induced apoptosis and cell death of PC3-AbiR and DU145-AbiR cells *in vitro*. A combination of QLD and abiraterone yielded a better tumor inhibition effect than QLD alone and abiraterone alone. Further investigation revealed that QLD restored the abiraterone sensitivity of PC3-AbiR and DU145-AbiR cells through modulating autophagy. These findings suggest that QLD might serve as a potential remedy to enhance the therapeutical effect of abiraterone for patients with castration-resistant prostate cancer.

## INTRODUCTION

According to Cancer Statistics in 2020, prostate cancer is the second leading cause of cancer-related mobility and mortality worldwide [1]. Advanced prostate cancer patients with cancer metastasis to other organs exhibit a low five-year survival rate and poor prognosis. Prior to early 2000s, only minimal chemotherapeutic agents (i.e., doxorubicin, cyclophosphamide, or epirubicin) were available for advanced prostate cancer treatment. However, they had been shown to offer miniature survival benefits [2, 3]. Since the approval for the use of chemohormonal agents (i.e., abiraterone acetate, enzalutamide) for metastatic prostate cancer, these seminal chemohormonal therapies reported significantly prolonged overall survival in men with advanced prostate cancer [4–6].

Abiraterone acetate is an inhibitor of androgen biosynthesis [7]. Since prostate cancer requires testosterone to grow, even in the other parts of the body, abiraterone acetate suppresses prostate cancer, especially castration-resistant prostate cancer, growth via reducing testosterone production. However, abiraterone acetate-resistance is inevitably developed after long-term use of abiraterone acetate [8]. Therefore, identifying new agents to overcome abiraterone acetate-resistance and enhance the treatment effect is critical for castration-resistant prostate cancer patients.

Chinese herbal medicine has been widely used in East Asia countries for the treatment of various diseases, including cancer [9–11]. Although the detailed molecular mechanism investigation is still in early-stage, the potent anti-tumor effect has been observed in a wide variety of herbal medicine, such as Curcumin, Resveratrol, Baicalein, Oleuropein, etc. [11–15]. Our group reported that Qi Ling decoction was capable of suppressing docetaxel resistance of prostate cancer through modulating miRNAs-mediated glycolysis in castration-resistant prostate cancer cells [16]. Moreover, we also found that Qi Ling decoction inhibited the invasion and migration gastric cancer cells, which the PI3K/Akt signaling may be involved in [17].

Inspired by these findings, we aim to investigate the synergistic effects of combined QLD and abiraterone on abiraterone-resistant prostate cancer and explore the underlying molecular mechanisms.

## RESULTS

### Establishment of abiraterone-resistant prostate cancer cell lines

**Figure 1:**
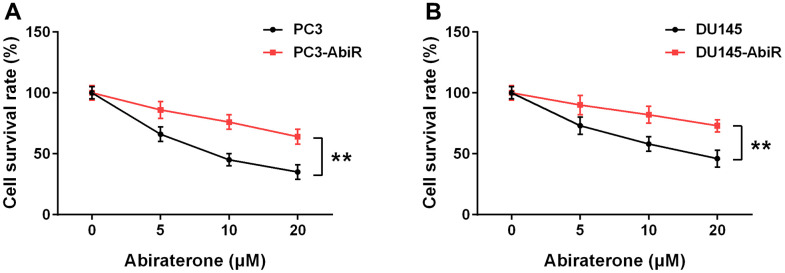
Resistance to abiraterone in PC3-AbiR (**A**) and DU145-AbiR (**B**) cells. Cells were treated with different concentrations of abiraterone for 48 hours, cell survival rate was calculated. Data were presented as mean ± SD. n=3. **p< 0.01.

To study the killing effect of abiraterone, PC3, PC3-AbiR, DU145, and DU145-AbiR cells were treated with different concentrations of abiraterone for 48 hours. Cell viability was measured using CCK-8 assay. As shown in Figure 1A, 1B, the cell survival rate of PC3-AbiR and DU145-AbiR was significantly higher than their counterparts (PC3, and DU145), confirming the successful establishment of abiraterone-resistant prostate cancer cell lines (PC3-AbiR and DU145-AbiR).

### QLD enhanced cytotoxic effect of abiraterone on PC3-AbiR and DU145-AbiR

**Figure 2:**
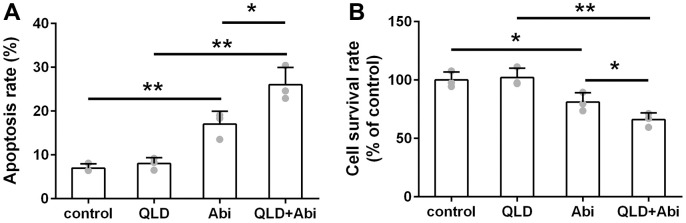
**QLD reduced abiraterone resistance ability of PC3-AbiR cells.** (**A**) The apoptotic cell death of PC3-AbiR cells was analyzed by FACS analysis and apoptosis rate was calculated. (**B**) Cell survival rate of PC3-AbiR cells in each group was detected by CCK8 kits. Data were presented as mean ± SD. n=3. *p< 0.05, **p< 0.01.

**Figure 3:**
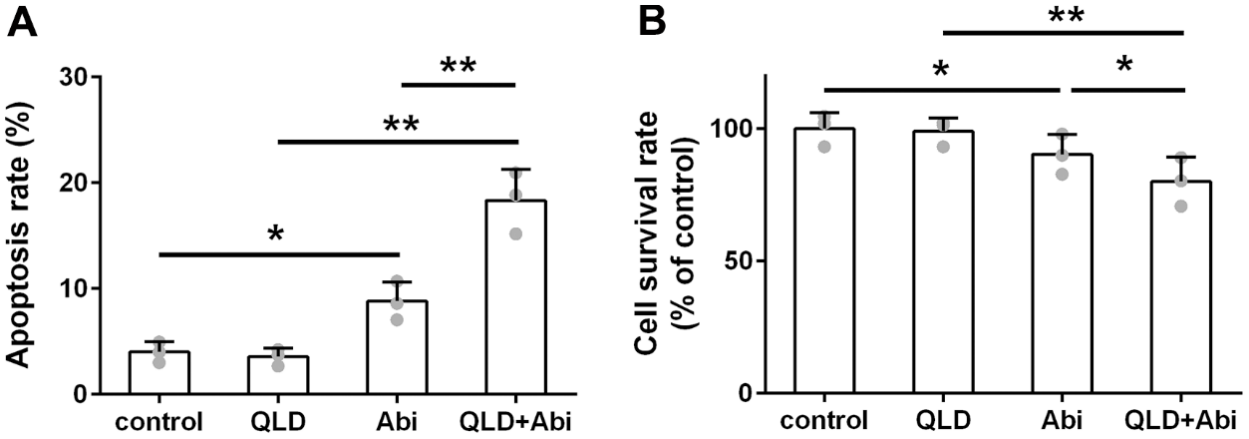
**QLD reduced abiraterone resistance ability of DU145-AbiR cells.** (**A**) The apoptotic cell death of DU145-AbiR cells was analyzed by FACS analysis and apoptosis rate was calculated. (**B**) Cell survival rate of PC3-AbiR cells in each group was detected by CCK8 kits. Data were presented as mean ± SD. n=3. *p< 0.05, **p< 0.01.

To address the question of whether QLD could decrease the abiraterone-resistant ability of PC3-AbiR and DU145-AbiR, these cell lines were treated with PBS (control), QLD, abiraterone (Abi), or combination of QLD and abiraterone (QLD+Abi) for 48 h. The cell apoptosis rate and survival rate were respectively determined by flow cytometry assay and CCK-8 assay. As illustrated in Figures 2A, 3A, QLD-alone had negligible cell killing effect, while Abi-alone led to increased apoptosis in PC3-AbiR and DU145-AbiR. Interestingly, QLD+Abi treatment resulted in more robust apoptosis in PC3-AbiR and DU145-AbiR. The cell survival results showed a similar trend as apoptosis results (Figures 2B, 3B), suggesting QLD enhanced the killing effect of Abi on PC3-AbiR and DU145-AbiR cells.

### QLD reduced abiraterone-induced autophagy in PC3-AbiR and DU145-AbiR

**Figure 4:**
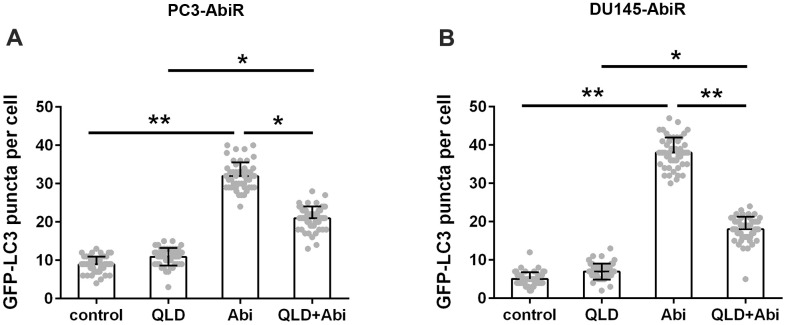
**QLD reversed abiraterone induced autophagy in PC3-AbiR and DU145-AbiR cells.** Autophagy was quantified by counting the GFP-LC3 puncta in the PC3-AbiR (**A**) and DU145-AbiR (**B**) cells. Data were presented as mean ± SD. n=3. *p< 0.05, **p< 0.01.

To clarify whether autophagy is involved in the synergistic effect of QLD+Abi on cell death, PC3-AbiR and DU145-AbiR cells were transfected with GFP-LC3 plasmid and exposed to control, QLD, Abi, or QLD+Abi treatment. The autophagosomes, represented as green (GFP) punctate signal, were examined under a fluorescent microscopy. The results in Figure 4A, 4B showed that the GFP-LC3 puncta were significantly enhanced in Abi group compared to the control or QLD group. The GFP-LC3 puncta in QLD+Abi group were also markedly higher than those in QLD group, but significantly lower than those in Abi group. These results suggested that QLD reduced autophagosome formation in Abi group.

### QLD decreased abiraterone-induced LC3-II and Becline 1 levels in PC3-AbiR and DU145-AbiR

**Figure 5:**
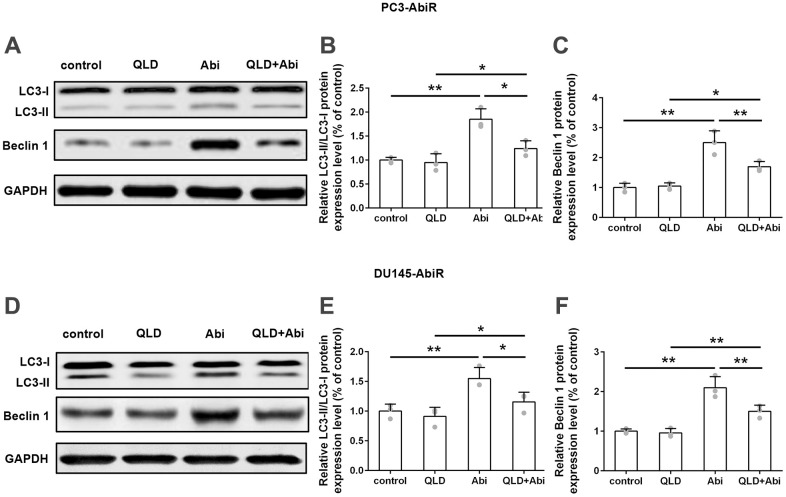
**QLD treatment significantly reduced LC3-II and Beclin 1 in PC3-AbiR and DU145-AbiR cells.** (**A**) Western blot was used to detect protein levels of LC3-II/ LC3-I and Beclin 1 in PC3-AbiR cells. (**B**, **C**) Relative protein expression was shown. (**D**) Western blot was used to detect protein levels of LC3-II/ LC3-I and Beclin 1 in DU145-AbiR cells. (**E**, **F**) Relative protein expression was shown. Data were presented as mean ± SD. n=3 for each group. *p< 0.05, **p< 0.01.

Next, PC3-AbiR and DU145-AbiR cells were treated with control, QLD, Abi, or QLD+Abi. The expression levels of LC3-I, LC3-II, Beclin 1, and GAPDH in those cells were determined by Western Blotting assay. As shown in Figure 5A–5F, Abi-exposure induced significant upregulation of the LC3-II/LC3-I ratio and Beclin 1 levels compared to control in PC3-AbiR and DU145-AbiR cells. The promotion effect of Abi on LC3-II/LC3-I ratio and Beclin 1 expression was partially restored by addition of QLD, confirming that QLD reduced Abi-induced autophagosome formation in PC3-AbiR and DU145-AbiR cells.

### QLD promoted anti-tumor effect of abiraterone on PC3-AbiR and DU145-AbiR

**Figure 6:**
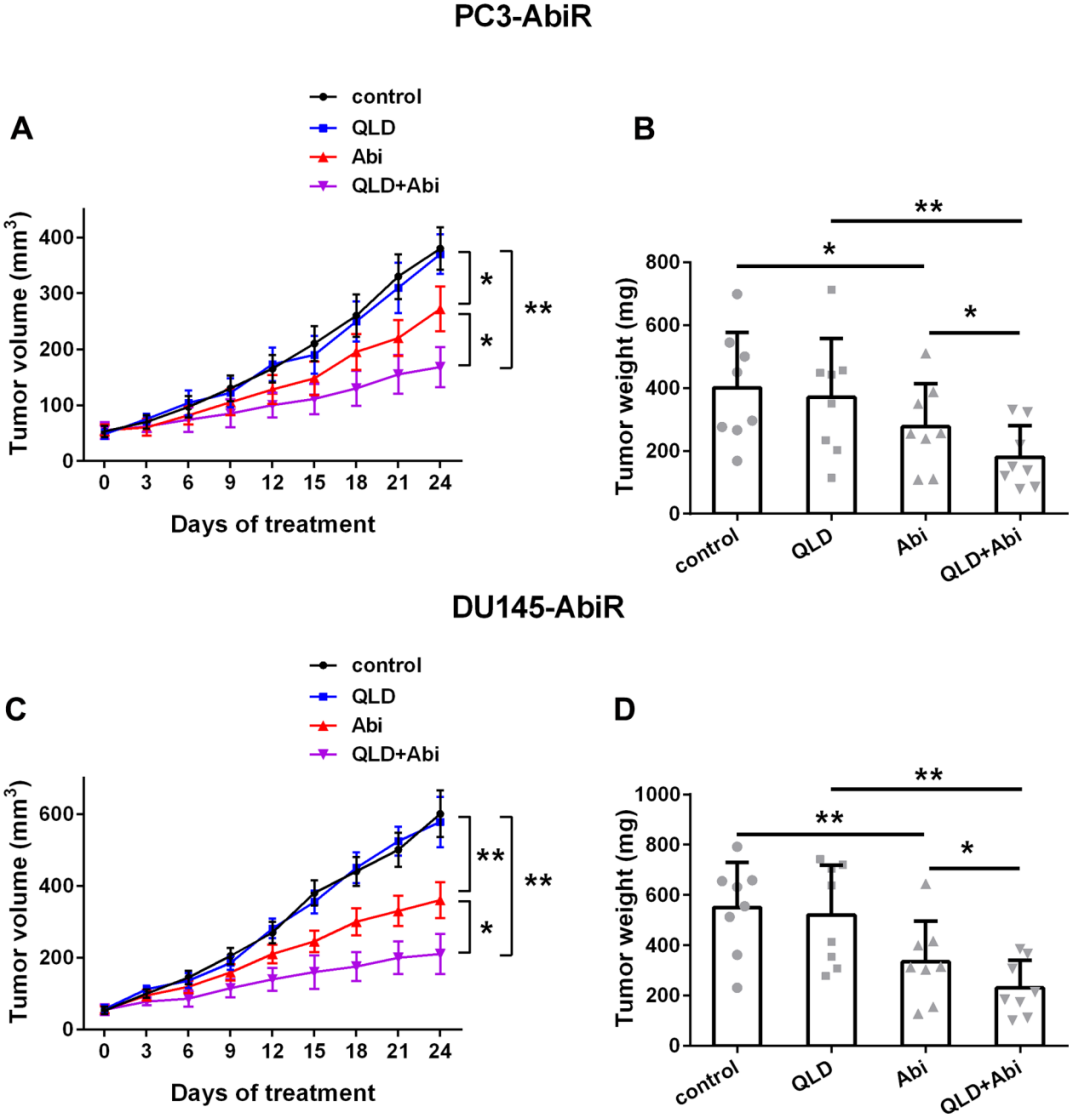
**QLD enhanced abiraterone treatment in mice.** The volume (**A**) and weight (**B**) of subcutaneous xenografts in each group in PC3-AbiR-bearing mice, and the volume (**C**) and weight (**D**) of subcutaneous xenografts in each group in DU145-AbiR-bearing mice were analyzed. Data were presented as mean ± SD. n=8 for each group. *p< 0.05, **p< 0.01.

Finally, to investigate the tumor inhibition effect of QLD+Abi on abiraterone-resistant prostate cancer cell lines, PC3-AbiR-, or DU145-AbiR-tumor bearing mice were treated with control, QLD, Abi, or QLD+Abi. As depicted in Figure 6A–6D, QLD-alone did not affect tumor growth, whereas Abi-alone significantly reduced tumor growth of PC3-AbiR and DU145-AbiR. Importantly, QLD and Abi combination treatment yielded the most profound tumor inhibition effect among four groups in both PC3-AbiR and DU145-AbiR tumor models, confirming that QLD was able to enhance the tumor growth inhibition effect of Abiraterone *in vivo*.

## DISCUSSION

QLD is a traditional Chinese medicine, which is extracted from various herbs, such as Kunshan, turmeric, raw astragalus, etc. Previous studies have demonstrated the potential anti-tumor effect of QLD in human cancers. For example, Chen et al., reported that QLD can inhibit cell migration and invasion of gastric cancer cells by blocking PI3K/AKT signaling pathway [17]. Clinical studies proved that QLD is able to decrease the recurrence of gastric cancer and improve the gastric cancer patients’ life quality. Similarly, Cao et al., showed that QLD enhanced the cytotoxic effect of docetaxel on docetaxel-resistant prostate cancer cells [12, 18]. Intriguingly, even single component extracted from one of eight herbs in QLD has shown anti-tumor effect. For instant, astragalus polysaccharide, extracted from Astragalus, is able to induce apoptosis of gastric cancer cells, and inhibit cell growth of breast cancer cells [19, 20]. Curcumin in turmeric has been proved to possess a variety of health benefits, including anti-tumor effect against breast, prostate, lung, and colon cancers [21–23]. Kushen injection, another Chinese medicine, has been used for cancer treatment in Chinese hospitals for over twenty years [24, 25]. In line with these findings, we reported that QLD-alone neither induce apoptosis, nor inhibit tumor growth of abiraterone-resistant prostate cancer cells, implying that QLD is also safe to healthy cells/tissues. Importantly, QLD significantly promoted anti-tumor effect of abiraterone on PC3-AbiR and DU145-AbiR cells as demonstrated by enhanced apoptosis rate *in vitro* and profound tumor growth inhibition *in vivo*.

Autophagy is a catabolic process that damaged organelles, useless proteins, or other cytoplasmic intrinsic or extrinsic components are encapsuled into a double-membrane vesicle and transported to the lysosome for degradation [26]. Cells can recycle a mass of molecules for rebuilding organelles while get rid of potentially harmful substances, including cancer drugs. Therefore, autophagy is vital for maintaining healthy cellular environment [27–29]. It is generally accepted that controlled levels of autophagy promote cell survival and overactivation of autophagy accelerates apoptosis [30]. In our study, we found that abiraterone treatment induced significantly upregulation of autophagosomes in PC3-AbiR and DU145-AbiR cells, implying these abiraterone-resistant prostate cancer cells can effectively eliminate abiraterone by sequestering abiraterone into autophagosomes and delivered to the lysosome for degradation. This ability confers PC3-AbiR and DU145-AbiR cells ability to resistant abiraterone. Of note, addition of QLD substantially reduced autophagosome formation in PC3-AbiR and DU145-AbiR cells with abiraterone treatment. Hence, we believed that QLD partially restored abiraterone sensitivity of PC3-AbiR and DU145-AbiR cells through inhibiting autophagy activity.

Although our finding suggest that QLD is able to promote abiraterone sensitivity in PC3-AbiR and DU145-AbiR cells, some unsolved questions deserved further investigation. For example, the detailed molecule mechanism of how QLD affects autophagosome formation is unknown. The dose-escalation study for QLD is urgently needed to be performed in various animal or clinical studies in order to support the clinical use of QLD in prostate cancer patients.

## CONCLUSIONS

In this study, we, for the first time, demonstrate that QLD is able to promote apoptosis and cell death in abiraterone-treated PC3-AbiR and DU145-AbiR cells *in vitro* and enhance the tumor inhibition effect of abiraterone on PC3-AbiR and DU145-AbiR cells *in vivo*. Further investigation reveals that QLD regulates the abiraterone sensitivity of PC3-AbiR and DU145-AbiR cells through modulating autophagy.

## MATERIALS AND METHODS

### Cell lines and reagents

PC3 and DU145 cell lines were purchased from ATCC (Manassas, VA, USA), and were cultured in DMEM medium with 10% fetal bovine serum (FBS, Hyclone, Logan, UT, USA). To establish abiraterone resistance prostate cancer cell lines, PC3 and DU145 cell lines were maintained in the increasing concentrations of abiraterone acetate (1 μM ~ 20 μM) over 12 months. The survived cells were recognized as abiraterone resistant cells and were further isolated. Those abiraterone resistant cell lines were referred to as PC3-AbiR and DU145-AbiR.

Qi Ling decoction (QLD) was prepared according to the publish paper [16].

### Cell Counting Kit-8 (CCK-8) assay

PC3, PC3-AbiR, DU145, and DU145-AbiR cell lines were treated with or without abiraterone acetate for 48 hours. Cell viability was assessed using CCK-8 assay (ab228554, Abcam, Shanghai, China) following manufactory’s instruction.

### Flow cytometry

PC3-AbiR, and DU145-AbiR cell lines were treated with phosphate-buffered saline (PBS), QLD, abiraterone acetate (Abi), or combination of QLD and Abi for 48 hours. Cells were stained with Annexin V labeled with CF Blue and propidium iodide (PI). After incubated in the dark room for 15 mins, cells were washed twice with cold PBS. Apoptotic cell population was assess using BD Accuri™ C6 Plus Flow Cytometer (Franklin Lakes, NJ, USA).

### Western blot

Protein samples were loaded on an 8% precast protein gels, and separated by SDS-PAGE. Subsequently, the separated proteins on the gel were transferred onto a polyvinylidene difluoride membrane. After blocked using a blocking buffer, the membrane was immersed in blocking buffer containing primary antibodies overnight. After incubation with secondary antibodies, the membrane was soaked in enhanced chemiluminescence (ECL). The protein signals were captured by a BioRad imaging system (ChemiDoc XRS+, Hercules, CA, USA). GAPDH was used as a control for normalization. Antibodies against LC3-I, LC3-II, Beclin 1, and GAPDH were purchased from Abcam (Shanghai, China).

### Animal experiments

Male BALB/c nude mice were purchased from the Vital River Laboratory Animal Technology Co. Ltd. (Beijing, China). 2×10^6^ PC3-AbiR or DU-145-AbiR cells were mixed with Matrigel (BD), and 100 μl tumor cell mixture was subcutaneously inoculated into the flanks of each mouse. After the tumor volume reached 50-100 mm3, PC3-AbiR or DU-145-AbiR tumor bearing mice were received oral administration of vehicle, QLD, abiraterone acetate (200 mg/kg), or combination of QLD and Abi. Tumor volume was measured at three days interval by formulation of Volume=1/2×length×width2. Mice were sacrificed 24 days post-tumor implantation. Tumor tissue from each mouse was collected and weighted.

### Statistical analysis

All data are presented as means ± standard deviation (SD). Difference between groups were assessed by one- or two-way ANOVA analysis followed by Bonferroni’s post hoc test. N number in the figure legends indicates biological replicates unless stated otherwise. P value less than 0.5 was considered as statistically significant.
